# Flexibility of the Prograamme of Spore Coat Formation in *Bacillus subtilis*: Bypass of CotE Requirement by Over-Production of CotH

**DOI:** 10.1371/journal.pone.0074949

**Published:** 2013-09-27

**Authors:** Rachele Isticato, Teja Sirec, Rosa Giglio, Loredana Baccigalupi, Giulia Rusciano, Giuseppe Pesce, Gianluigi Zito, Antonio Sasso, Maurilio De Felice, Ezio Ricca

**Affiliations:** 1 Department of Biology, Federico II University, Naples, Italy; 2 Department of Physics, Federico II University, Naples, Italy; Loyola University Medical Center, United States of America

## Abstract

Bacterial spores are surrounded by the coat, a multilayered shell that contributes in protecting the genome during stress conditions. In *Bacillus subtilis*, the model organism for spore formers, the coat is composed by about seventy different proteins, organized into four layers by the action of several regulatory proteins. A major component of this regulatory network, CotE, is needed to assemble the outer coat and develop spores fully resistant to lysozyme and able to germinate efficiently. Another regulator, CotH, is controlled by CotE and is present in low amounts both during sporulation and in mature spores. In spite of this CotH controls the assembly of at least nine outer coat proteins and cooperates with CotE in producing fully resistant and efficiently germinating spores. In order to improve our understanding of CotH role in spore formation, we over-produced CotH by placing its coding region under the control of a promoter stronger than its own promoter but with a similar timing of activity during sporulation. Over-production of CotH in an otherwise wild type strain did not cause any major effect, whereas in a *cotE* null background a partial recovery of the phenotypes associated to the *cotE* null mutation was observed. Western blot, fluorescence microscopy and Surface-Enhanced Raman Scattering spectroscopy data indicate that, in the absence of CotE, over-production of CotH allowed the formation of spores overall resembling wild type spores and carrying in their coat some CotE−/CotH-dependant proteins. Our results suggest that the *B. subtilis* spore differentiation programme is flexible, and that an increase in the amount of a regulatory protein can replace a missing partner and partially substitute its function in the assembly of the spore coat.

## Introduction

The bacterial endospore (spore) is a dormant cell, resistant to harsh conditions and able to survive extreme environmental conditions [1]. Bacteria of the *Bacillus* and *Clostridium* genera produce the spore within a sporangium, formed by a small cell, the forespore, carried inside a big cell, the mother cell. Through a series of maturation events, that require the contribution of both cells, the forespore will become the mature spore, that will be released at the end of the sporulation cycle by the lysis of the mother cell [2], [3]. In *Bacillus subtilis,* the model system for spore formers, resistance of the spore to noxious chemicals, lytic enzymes and predation by soil protozoans is mainly due to the coat, a complex, multilayered structure of more than 70 proteins that encases the spore [4], [5], [6]. Proteins that constitute the coat are produced in the mother cell and deposited around the outer membrane surface of the forespore in an ordered manner [5], [6]. A small subset of coat proteins have a regulatory role on the formation of the coat (for a recent review see McKenney et al. [6]). Those proteins, referred to as morphogenic factors, do not affect the synthesis of the coat proteins but control their correct assembly outside of the outer forespore membrane [5], [6]. Within this subset of regulatory proteins, CotE plays a crucial role [7]. It forms a ring-like structure that surrounds the forming spore and drives the formation of the outermost layers, the outer coat and the recently identified crust [6], [8]. However, not all CotE molecules are assembled into the ring-like structure and CotE molecules are also found in the mother cell cytoplasm, at least up to eight hours after the start of sporulation [9]. CotE was first identified as a morphogenic factor in a seminal study in which an ultrastructural analysis indicated that a *cotE* null mutation prevented formation of the electron-dense outer layer of the coat while did not affect inner coat formation [8]. Such structural defects result in spores with a strongly reduced efficiency of germination and sensitive to lysozyme [8].

An additional regulatory protein found in the *B. subtilis* coat is CotH, known to be controlled by CotE and to control the assembly of a subset of at least nine outer coat proteins [10]. Therefore, in a hierarchical way, CotE controls CotH which in turn regulates nine outer coat proteins [10]. CotH structural gene, *cotH,* is transcribed by the sporulation-specific sigma factor of the RNA polymerase σ^K^ and is negatively controlled by the transcriptional regulator GerE [11]. As a consequence, transcription of the gene is induced 4 hours after the beginning of sporulation, peaks one hour later and then rapidly decreases [11]. It has been recently observed that the *cotH* promoter is located 812 nucleotides upstream of the coding region. The long sequence at 5′ end of the *cotH* gene is not translated and completely overlaps the divergent *cotG* gene, coding for an abundant CotE/CotH-dependent outer coat protein [12]. A mutant lacking CotH produces spores altered in both the inner and the outer layer of the coat, leading to the hypothesis that CotH is localized at the interface between the two coat layers [13]. While spores of a strain lacking CotH have as their only phenotype a slightly reduced efficiency of germination, spores of a double mutant lacking CotE and CotH are strongly impaired in the efficiency of germination and in the resistance to lysozyme [14]. Those phenotypes are more severe in spores of the double mutant strain than in spores lacking only CotE, suggesting that CotH contributes with CotE in assembling a normal spore, resistant to lytic enzymes and able to germinate efficiently [14].

Here we observed that the CotH protein has a short half-life and its concentration drops soon after that transcription of the structural gene has been turned off. In spite of the short half-life within the cell CotH controls the assembly of other nine coat proteins. To understand in more details the mechanism of CotH action during spore coat formation we constructed a mutant strain over-producing CotH and analyzed its phenotype. Our data indicate that over-production of CotH partially bypasses the requirement of CotE for coat formation.

## Results and Discussion

### CotH is Present in the Sporulating Cell for a Short Time during Sporulation

**Figure 1:**
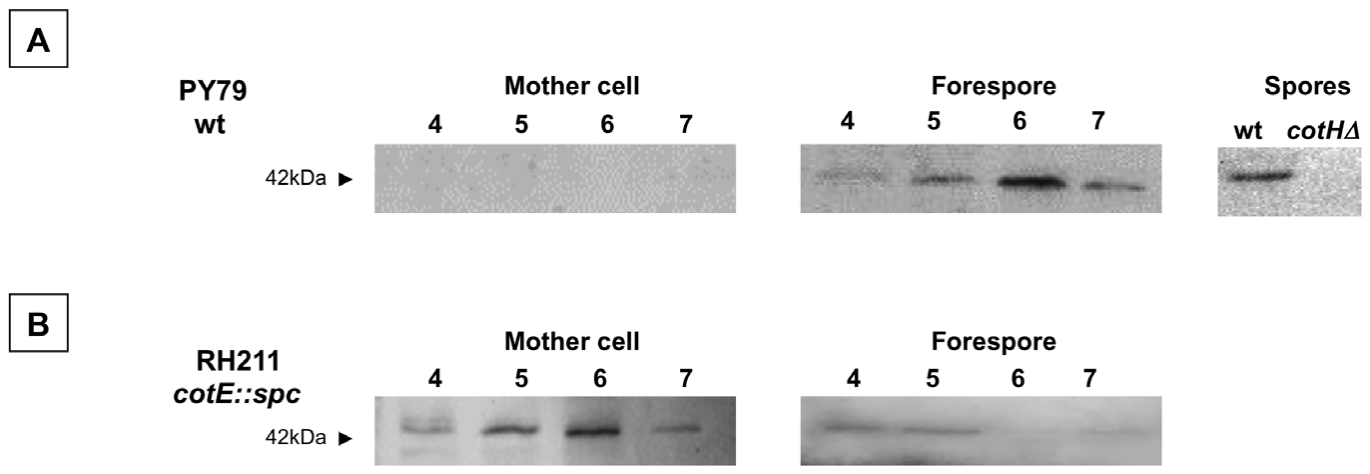
CotH in sporulating cells of a wild type and an isogenic *cotE* null mutant. Proteins extracted from sporulating cells (forespores and mother cells) at various times during sporulation and from mature spores of a wild type (A) or a mutant strain lacking CotE (B). Proteins were fractionated on 15% polyacrylamide gels, electrotransferred to membranes and reacted with the anti-CotH antibody.

A western blotting approach was used to analyze the presence of CotH in sporulating cells. Proteins were extracted from forespore or mother cell compartments of the sporangia at various times during sporulation and analyzed by western blot with anti-CotH antibody [12]. In sporulating cells CotH was found only in the forespore fraction indicating that it is assembled around the forming spore right after its synthesis and does not accumulate in the mother cell compartment (Fig. 1A). CotH was detected starting four hours after the onset the sporulation (T4), reached its maximal concentration two hours later (T6) and then its amount decreased (Fig. 1A). As a consequence of that decrease only minimal amounts of CotH could be extracted from mature spores. Indeed, a CotH-specific protein could be detected only when 30 µg of total proteins extracted from 18 hours-old spores were fractionated (Fig. 1A). In a *cotE* mutant, in which CotH assembly is impaired [13], CotH mostly accumulated in the mother cell (Fig. 1B). As in the wild type, CotH reached its maximal amount at T6 and then drastically decreased (Fig. 1B). A possible explanation of such decrease is that CotH becomes insoluble because of putative post-translational modifications occurring in the sporulating cell. However, when the insoluble coat protein fraction was extracted, as previously described [15], and analyzed by western blot with anti-CotH antibody, CotH was not found (Fig. S1 in Supplementary Material), suggesting that it does not become insoluble. A potential alternative explanation is that CotH is degraded due either to an intrinsic instability or to a putative unknown protease. Either way, in spite of its short half-life, CotH is needed to allow the assembly of at least nine outer coat proteins [10].

### Expression of *cotH* Under the Control of the *cotA* Promoter

**Figure 2:**
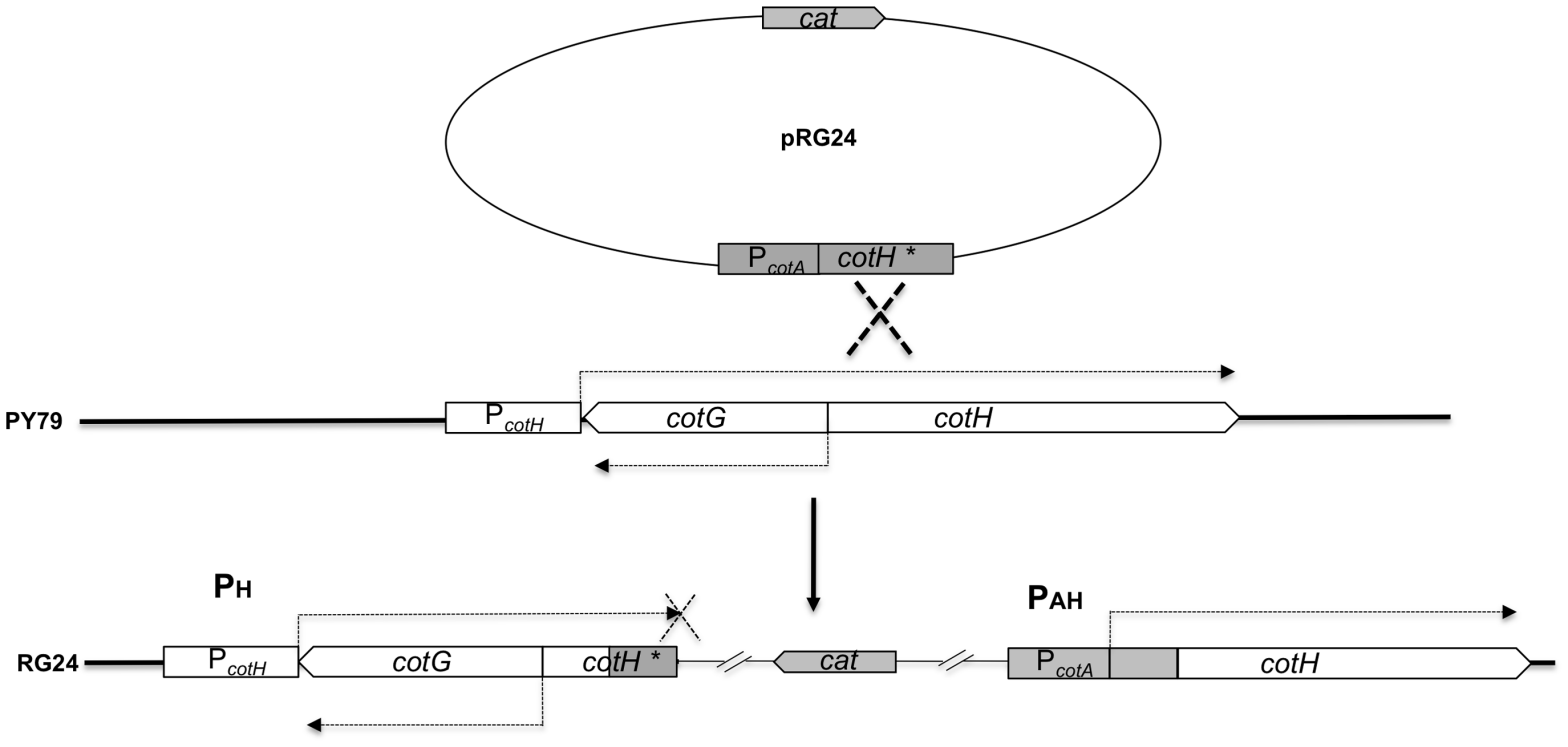
Construction of the RG24 strain. Plasmid pRG24 carries the 5′ region of the *cotH* coding region posed under the transcriptional and translational control of the *cotA* promoter. The non-replicative plasmid was used to transform competent cells of the wild type strain PY79. Chloramphenicol-resistant clones originated by a single cross-over event between homologous DNA regions on the plasmid and on the chromosome. In the resulting strain, RG24, the *cotH* promoter (P_H_) transcribes a truncated form of *cotH* while an entire gene is transcribed by the *cotA* promoter (P_AH_).

**Figure 3:**
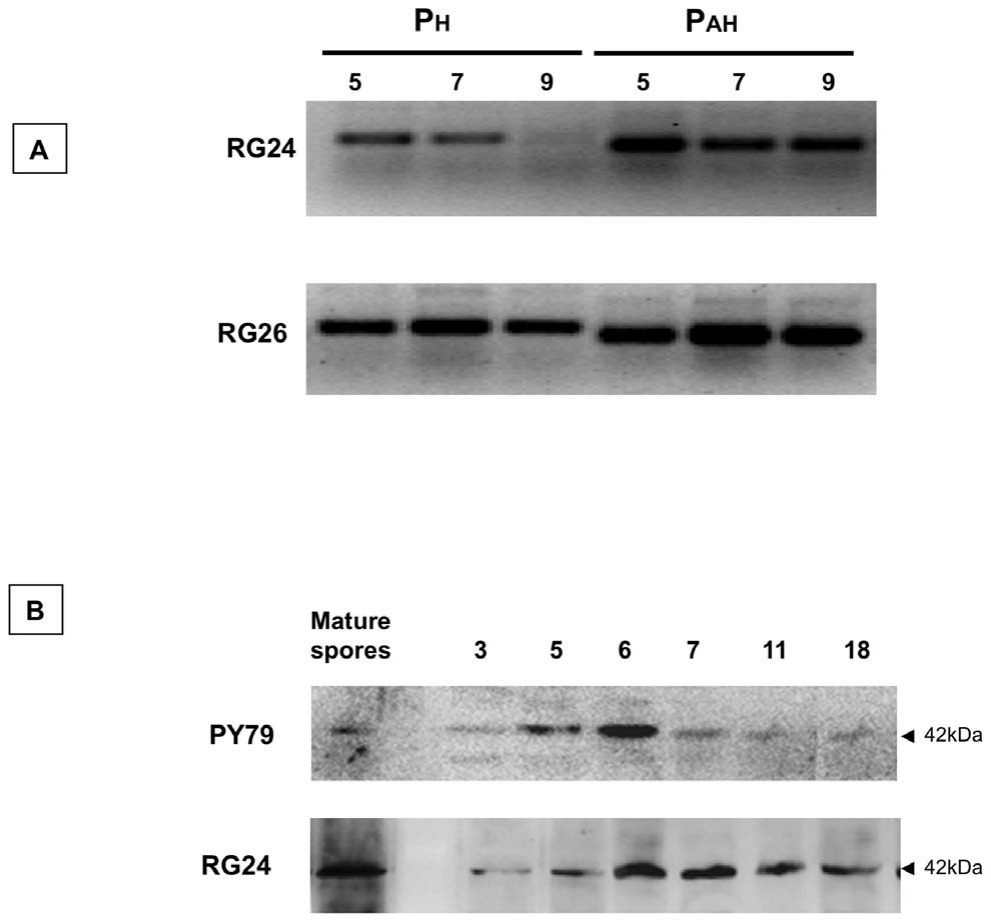
*cotH* expression in RG24. (A) RT-PCR experiments were used to analyze *cotH* transcription in RG24 and in RG26, a *gerE* derivative of RG24. Total RNA extracted from the same sporulating cells at different times during sporulation was used to obtain cDNA and perform the PCR analysis for P_H_ and P_AH_ (see Fig. 2). (B) Western blot analysis with anti-CotH antibody of proteins extracted from mature spores and sporulating cells at various times during sporulation of strain RG24 and its isogenic wild type strain PY79. For sporulating cells the entire sporangium was used to extract proteins. Proteins were fractionated on 15% polyacrylamide gels, electrotransferred to membranes and reacted with the anti-CotH antibody.

To elucidate the role of CotH in spore coat morphogenesis we decided to construct a mutant in which CotH was more abundant than in the wild type. To this aim we decided to clone the *cotH* coding region under the transcriptional and translational signals of the *cotA* gene. *cotA* is transcribed by Sigma K but its promoter is more efficient than the *cotH* promoter in initiating transcription [16]. The *cotA* promoter was fused to a DNA fragment containing the 5′ region of the coding part of *cotH* (Fig. 2). The gene fusion, carried by the integrative plasmid pRG24, was used to transform competent cells of a wild type strain of *B. subtilis,* PY79. By a single (Campbell-like) cross-over recombination event between homologous DNA regions present on the plasmid and on the chromosome, the *cotA* promoter was inserted upstream of an entire *cotH* coding part (we will herein refer to it as P_AH_) while the *cotH* promoter was driving the transctiption of a truncated *cotH* gene (herein P_H_) (Fig. 2). Transcription from P_AH_ and P_H_ in the same strain (RG24) was monitored by RT-PCR during sporulation. As shown in Fig. 3A, transcription from P_AH_ was significantly more efficient than from P_H_ at all time points analyzed. In particular, at T9 transcription was totally abolished from P_H_ and still active from P_AH_ (Fig. 3A). In strain RG26, an isogenic derivative of RG24 lacking *gerE*, transcription due to both promoters was increased with respect to RG24. This result was expected since both promoters are known to be negatively regulated by GerE [14], [17] and confirmed that the DNA region containing the *cotA* promoter used to construct strain RG24 also contained its entire regulatory region.

Strain RG24 was then used to monitor by western blot with anti-CotH antibody the presence of CotH in sporulating cells and in mature spores. As shown in Fig. 3B, sporulating cells and mature spores of strain RG24 contained higher levels of CotH than the wild type strain (PY79) at all time points analyzed. Therefore, the increase in the efficiency of transcription of *cotH* prolonged the presence of CotH in the sporulating cells and increased CotH amount around forespores and in mature spores.

### Over-production of CotH Partially Bypasses the *cotE* Phenotypes

**Figure 4:**
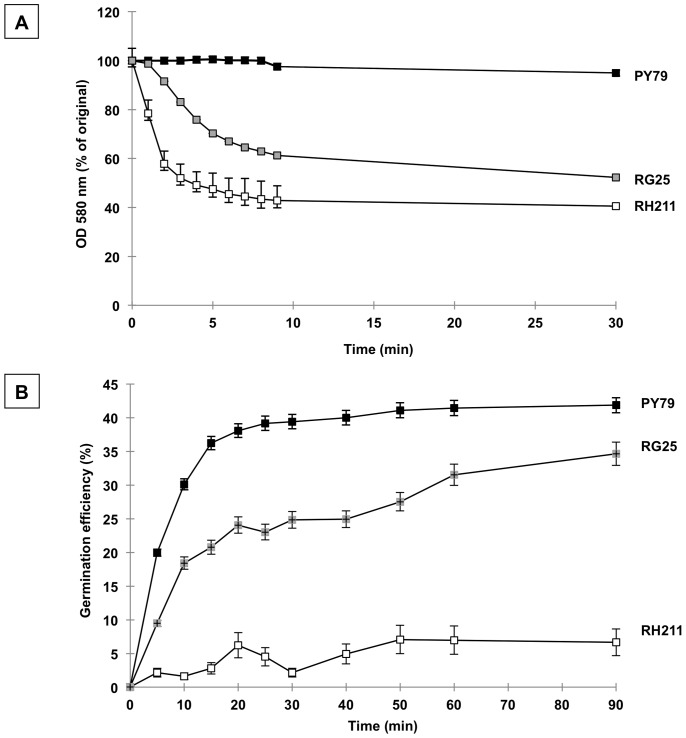
Bypass of *cotE* phenotypes by CotH over-production. Effect of lysozyme (A) and germination efficiency (B) of spores of the wild type (closed squares), a *cotE* null mutant (open squares) and a *cotE* null mutant over-producing CotH (gray squares). OD, optical density. Germination was induced by Asn-GFK as previously reported [14]. Error bars are based on the standard deviation of 5 independent experiments.

To investigate the effects of CotH over-production we analyzed the efficiency of sporulation, the resistance to chemicals and lytic enzymes and the efficiency of germination of spores of strain RG24. Those spores were identical to isogenic wild type (PY79) spores for all phenotypes analyzed (not shown), indicating that CotH over-expression did not affect spore function in an otherwise wild type strain. However, when CotH was over-produced in the isogenic mutant RG25, lacking CotE, we observed a partial suppression of the typical *cotE* phenotypes: sensitivity to lysozyme (Fig. 4A) and reduced efficiency of germination (Fig. 4B). The sensitivity to lysozyme was also measured by determining the colony forming units (CFU). Compared with cells not treated with lysozyme, the wild type cells (PY79) showed a 30% reduction of CFU after lysozyme treatment (Methods) while the *cotE* mutant (RH211) showed a much stronger effect (92% reduction of viability). RG25 cells showed a CFU reduction of 62%, confirming the partial suppression of the *cotE* phenotype observed in Fig. 4A. A previous report [14] showed that spores of a double *cotE cotH* null mutant have an increased sensitivity to lysozyme and a reduced efficiency of germination than a single *cotE* null mutant. This observatiom suggested that CotH cooperates with CotE in determining the resistance to lysozyme and the efficiency of germination. Now, we observe that the increased and prolonged presence of CotH partially restores the resistance to lysozyme and the germination efficiency lost because of the *cotE* null mutation.

**Figure 5:**
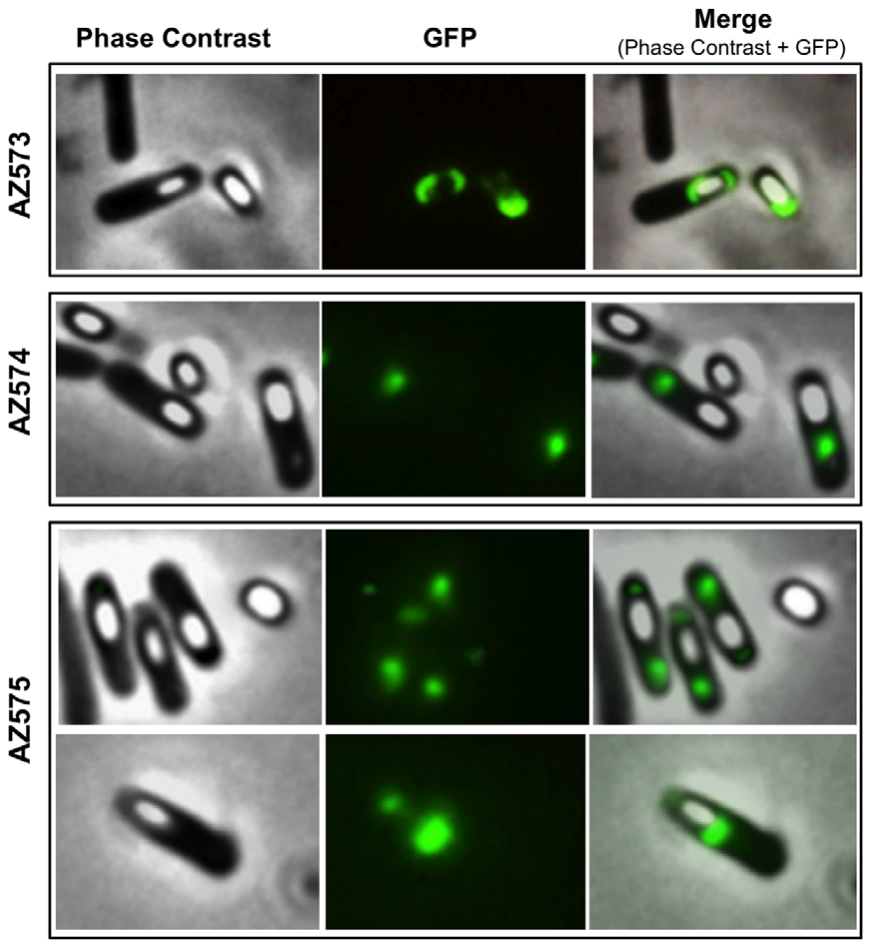
Fluorescence microscopy of sporulating cells and spores of a *cotZ::gfp* strain. A *cotZ::gfp* fusion (Methods) was introduced into an otherwise wild type strain (AZ573) and into isogenic strains lacking CotE (AZ574) or over-producing CotH in the absence of CotE (AZ575). Representative microscopy fields are shown by phase contrast and fluorescence (GFP) microscopy. Panels on the right are the merge of contrast phase and fluorescence images. Cultures were grown in DSM for 24 hours.

It has been recently observed that CotE is essential for the formation of the crust, the outermost layer of the *B. subtilis* coat [6]. In order to study the effects of CotH over-production on this additional *cotE* phenotype, we analyzed by fluorescence microscopy sporulating cells carrying a CotZ-GFP fusion. CotZ has been identified as a crust component and its assembly is an indirect evidence of crust formation [6]. As shown in Fig. 5, in wild type spores (AZ573) CotZ was localized at the poles of forespores and free spores, while in strain AZ574, in the absence of CotE, the fluorescence due to CotZ was in the mother cell cytoplasm and never associated to forespore or mature spores. In strain AZ575, a derivative of RG25 carrying the *cotZ::gfp* gene fusion, CotZ localized in proximity of one or both poles of forespores (Fig. 5). In this strain fluorescence was never associated to free spores, suggesting that proper crust formation also requires CotH-independent proteins. All together our results indicate that CotH can partially replace CotE in driving the formation of a functional outer coat.

### Over-production of CotH Bypasses the CotE Requirement for the Assembly of CotH and CotH-dependent Outer Coat Proteins

**Figure 6:**
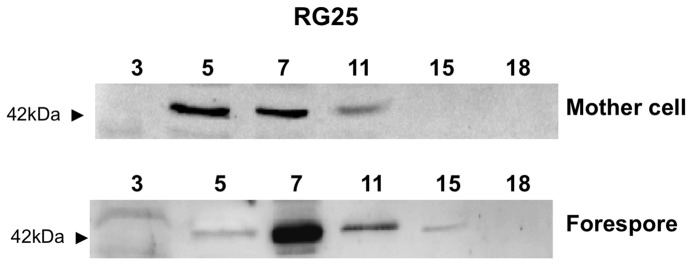
Assembly of CotH in the absence of CotE. Proteins were extracted from mother cells or forespores of sporulating cells of strain RG25, fractionated on 15% SDS-PAGE, electrotrasferred on a membrane and reacted against anti-CotH antibody.

In a *cotE* mutant the entire outer coat is not formed [7] and the assembly of CotH within the coat is prevented (Fig. 1B) [13]. To understand how over-production of CotH partially bypassed the CotE requirement for the formation of a functional outer coat we first analyzed the cell fate of the over-produced CotH molecules. Proteins were extracted from forespore or mother cell compartments of the RG25 strain at various times during sporulation and analyzed by western blot with anti-CotH antibody. As shown in Fig. 6, CotH was found in both compartments between T5 and T11, and was also found in very low amounts at T15 only in the forespore. Therefore, when over-produced, CotH can be assembled around the forming spore in the absence of CotE. However, CotH was never observed in mature spores of strain RG25 (not shown), suggesting that in the absence of CotE, CotH localizes around the forming spore but is not assembled within the coat structure.

**Figure 7:**
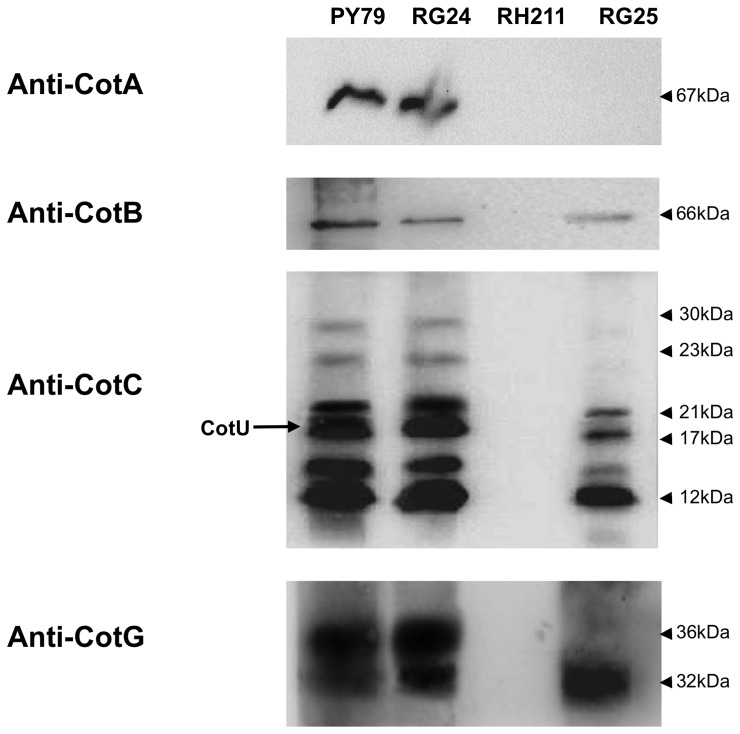
Assembly of CotE-dependent proteins. Proteins extracted from mature spores of a wild type strain or from isogenic strains over-producing CotH (RG24), lacking CotE (*cotE*) and over-producing CotH in the absence of CotE (RG25) were probed with anti-CotA, anti-CotB, anti-CotC and anti-CotG antibodies. Proteins were fractionated on 15% polyacrylamide gels, electrotransferred to membranes and reacted with the indicated antibodies.

**Figure 8:**
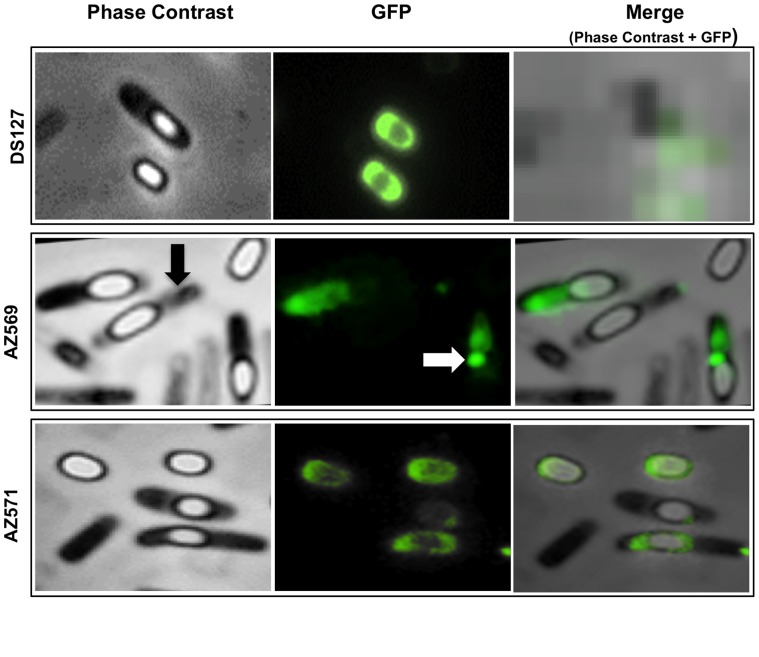
Fluorescence microscopy of sporulating cells and spores of a *cotC::gfp* strain. A functional *cotC::gfp* fusion [22] (DS127) was introduced into isogenic strains lacking CotE (AZ569) or over-producing CotH in the absence of CotE (AZ571). A representative microscopy fields for each strain is shown by phase contrast and fluorescence (GFP) microscopy. Panels on the right are the merge of contrast phase and fluorescence images. Black and white arrows point to a late sporulating cell containing an almost mature spore and a fluorescent spot probably associated to the spore surface, respectively. Cultures were grown in DSM for 24 hours.

Next we analyzed the coat assembly of various CotE-dependent coat proteins in mature spores of the *cotE* mutant RH211 and in the isogenic mutant RG25 lacking CotE but over-expressing CotH. As a control we also analyzed strain RG24 over-producing CotH in an otherwise wild type strain. In all cases analyzed, over-production of CotH in a wild type strain did not affect the assembly of other coat proteins while in the absence of CotE it allowed the assembly of some CotE-dependent outer coat proteins (Fig. 7). In particular, over-production of CotH affected the assembly of the CotE−/CotH-dependent CotB, CotC, CotG and CotU proteins but not of the assembly of CotA that is CotE-dependent and CotH-independent (Fig. 7). In the case of CotG, we observed that two CotG forms of 32 and 36 kDa were present in the coat of wild type and RG24 spores, while in the coat of RG25 spores only the 32 kDa protein was found (Fig. 7). Although other experiments are needed to clarify this point, we speculate that the 36 kDa is a post-translationally modified form of CotG and that such modification requires a still unknown factor, absent in strain RG25. CotG is known to mediate the conversion of the 46 kDa form of CotB to the 66 kDa, mature form [18]. The presence of the 66 kDa form of CotB in spores of strain RG25 (Fig. 7) indicates that the 32 kDa form of CotG alone is able to drive CotB maturation. In the case of CotC, six specific proteins were recognized by anti-CotC antibody in wild type and RG24 spores. Of those proteins the 17 kDa one is the CotC-homolog CotU [19], that is assembled within the coat of strain RG25, as the other CotE-CotH-dependent proteins analyzed (Fig. 7). The two CotC forms of 23 and 30 kDa, present in spores of wild type and RG24 strains were not found in spores of strain RG25. The 23 kDa protein is known to be a CotC-CotU heterodimer and is known to be formed only when CotE directly contacts CotC and CotU [20]. Therefore, it is not surprising that this protein is not formed in strain RG25, lacking CotE (Fig. 7). The 30 kDa protein is probably the result of post-translational modification of CotC occurring on the spore coat [21] and, as in the case of the 36 kDa form of CotG, its formation probably requires a putative unknown factor absent in strain RG25. However, the lack of the 23 and 30 kDa CotC forms did not affect the overall assembly of CotC. A fluorescence microscopy analysis of sporulating cells carrying a CotC-GFP fusion [22] showed that while in wild type spores CotC was localized around forespores and mature, free spores, in strain AZ569, in the absence of CotE the fluorescence due to CotC was rather diffuse in the mother cell cytoplasm. Fluorescence by some sporulating cells could be observed at one pole of the forespore (see white arrow in Fig. 8), but never around mature spores or forespores at late stages of maturation (with mother cell close to lyse; see black arrow in Fig. 8). In strain AZ571, a derivative of RG25 carrying the *cotC::gfp* gene fusion, the absence of CotE was bypassed by CotH over-production and CotC localized around forespore and free spores (Fig. 8). As a control, sporulating cells of a strain carrying a CotA-GFP fusion were also analyzed by fluorescence microscopy. In agreement with data of Fig. 7, CotA failed to assemble around the forming spore in the absence of CotE and over-production of CotH did not have any effect on CotA recruitment (Fig. S2 in Supplementary Material). Results of Fig. 7 and 8 then indicate that over-production of CotH bypasses the requirement of CotE for the assembly of the CotE−/CotH-dependent coat proteins CotB, CotC, CotG and CotU.

### Over-production of CotH Bypasses the CotE Requirement for Spore Coat Formation

**Figure 9:**
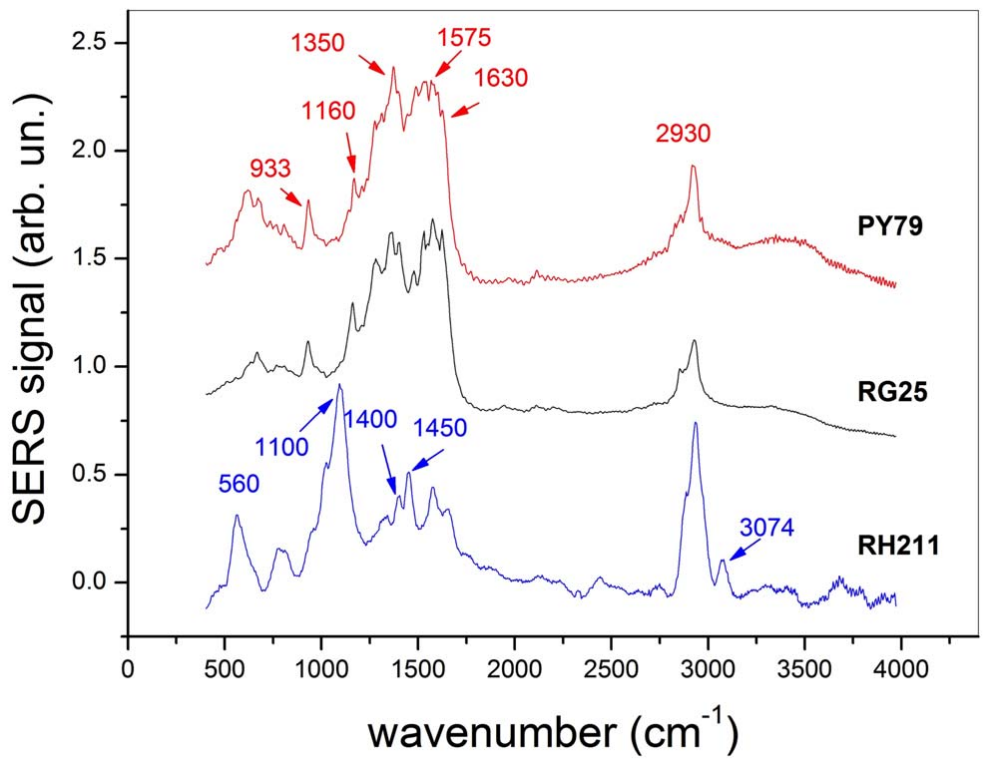
Surface-Enhanced Raman Spectroscopy analysis. Normalized SERS spectra, in the region between 400 and 3000-1, of wild-type spores (trace a), spores over-producing CotH and lacking CotE (trace b) and spores lacking CotE (trace c). Each trace results by the average of 30 different spectra from single spores.

To obtain a better understanding of the effects of the over-production of CotH in a strain lacking CotE on the overall coat structure, we performed a SERS (Surface Enhanced Raman Scattering) spectroscopy analysis. With respect to the basic Raman technique, a SERS approach provides two main advantages: i) it allows a high enhancement of the Raman efficiency, in principle even of 8–10 orders of magnitude, making this technique suitable for the detection of molecules at low concentration; ii) in comparison with Raman spectroscopy, it is much more surface-sensitive because it is based on the interaction of molecules in close proximity (in nanometer scale) of the metallic substrates [23]. Both these features arise from the excitation of localized plasmonic resonances induced in metallic nano-structures when they are irradiated by a properly selected laser wavelength. The result is a strong enhancement of the electromagnetic field close to the metallic surface and, hence, a dramatic increase of the Raman signal. Therefore, SERS is a powerful tool to analyze the structure of the surface of an object without the interference of the bulk response. Figure 8 shows the SERS spectra, in the region between 400 and 3000 cm^−1^, of wild-type PY79 spores (red trace), of RH211 spores (lacking CotE) (blue trace) and of spores of strain RG25 over-producing CotH in the absence of CotE (grey trace). In order to increase the signal-to-noise ratio, each trace of Figure 9 is the mean of 30 different spectra of single spores for each of the three strains. Moreover, each spectrum was normalized to the height of the most prominent peak. The analysis of Fig. 9 shows that PY79 and RG25 spectra have evident similarities, while the RH211 spectrum significantly differs from both PY79 and RG25. In particular, PY79 and RG25 spectra exhibit pronounced bands in the region around 1350 and 1575 cm^−1^, clearly arising from the convolution of many different slightly shifted peaks. Furthermore, sharp features of PY79 and RG25 spectra appear also around 933, 1160, 1630 and 2930 cm^−1^, as well as less intense bands around 672, 811 and 2100 cm^−1^. In contrast, the RH211 spectrum exhibits features around 560, 1100, 1400, 1450, and 3074 cm^−1^, with the latter three assignable to dipicolinic acid (DPA), probably released by the mutant spores. In the region between 1350 and 1575 cm^−1^ the RG25 spectrum has peaks sharper than the PY79 spectrum, suggestive of a larger molecular variety in the latter sample. This conclusion is also supported by the presence of additional peaks in the low-frequency regions (see, for instance, features around 618 and 735 cm-1) of the PY79 spectrum. Finally, peaks around 1575 and 2930 are similar in the three samples. Assignment of SERS peaks is not an easy task, in particular when biological samples are analyzed. This is mainly due to a large variety of molecules contributing to the spectra and the possible spectral shift introduced by SERS mechanism [23]. The latter depending on the environment of the scattering molecules that adds a further degree of freedom. Nevertheless, some important information can be gained: i) spectra PY79 and RG25 are clearly similar to each other; ii) sample PY79 has a larger molecular variety than the RG25 sample. Those conclusions are both consistent with previous data showing a phenotypic bypass of CotE requirements due to CotH over-production (Figs. 4–5) and the presence of a reduced molecular variety in spores of the RG25 strain due to the lack of CotE-dependent, CotH-independent coat proteins (Fig. 7).

## Conclusions

A first conclusion of this work is that the CotH protein has a short half-life being found only around the forming spore between 5 and 7 hours after the onset of sporulation. Soon after that its structural gene is no longer expressed the amount of CotH drastically decreases. In spite of the short half-life, CotH controls the assembly within the coat of a subset of CotE-dependent proteins and contributes with CotE in developing spores fully resistant to lysozyme and able to germinate. Such cooperation is somehow independent of the action of the subset of CotE−/CotH-dependent proteins since a double mutant lacking CotE and CotH is less resistant to lysozyme and less able to germinate than a mutant lacking only CotE.

A second, main conclusion of this work is that production of high amounts of CotH does not affect otherwise wild type spores but allows a partial suppression of CotE requirement for spore coat formation. In the absence of CotE, over-production of CotH allows the formation of almost wild type spores: i) CotH is found around the forming spore; ii) at least four CotE−/CotH-dependent proteins are assembled within the coat; iii) SERS data indicate that the overall spore composition is more similar to wild type than to *cotE* spores; iv) like CotA, other CotE-dependent CotH-independent proteins are most probably not assembled, originating a coat with a reduced molecular variety; and v) the typical *cotE* phenotypes are bypassed with spores able to survive lysozyme and germinate. These results allow us to hypothesize that CotH assembles several proteins onto the coat only after its recruitment to the spore surface. According to this model, CotE increses the concentration of CotH at the spore surface, indirectly allowing the assembly of several CotH-dependent coat proteins. If CotH is over-produced CotE is no longer required and CotH-dependent proteins can be assembled in a *cotE* mutant. A future challenge raising from this work will be to analyze in details the interaction between CotE and CotH to verify whether other proteins are involved.

These conclusions also suggest that in *B. subtilis* the differentiation programme driving to coat formation is somehow flexible, with a regulatory protein that can replace a missing partner and partially substitute its function. This can be viewed as a protection for the species, allowing the formation of an almost wild type spore when a key gene is either mutated or not efficiently expressed. We speculate that the artificial situation due to the deletion of the *cotE* gene and the over-production of CotH may mimic what happens in other spore formers. Indeed, while CotE has been found in all *Bacillus* species so far analyzed, it has never been found in *Clostridia* [5]. Interestingly, some *Clostridium* species (*C. perfringens* ATCC13124, *C. thermocellum* ATCC27405, *C. cellulolyticum* H10 and *C. difficile* QCD-32g58) have a CotH homolog [5]. The study of the coat structure of those spore formers will likely contribute new insights on the role of the various regulatory proteins and will be an additional interesting future challenge arising from this work.

## Materials and Methods

### Genetic and Molecular Procedures

Isolation of plasmids, restriction digestion, and ligation of DNA were carried out by standard methods [24]. Chromosomal DNA from strain PY79 [25] *B. subtilis* was isolated as described elsewhere [26]. RG24 strain was obtained by fusing the *cotA* promoter to a DNA fragment containing the 5′ 850 bp of *cotH* coding part. This fusion was obtained by using the gene splicing by overlap extension (SOEing) technique [27]. Briefly, two PCR products were obtained with oligonucleotide pairs PCotAs and CotA3anti (to amplify the *cotA* promoter region of 577 bp) and H32s/H13 (amplifying the 850 bp fragment starting from the ATG to the unique *Eco*RI site internal of *cotH* coding sequence) (Suppl. Mat. Table S1). The obtained products were used as templates to prime a third PCR with the external primers CotApS and H13 (Suppl. Mat. Table S1). The modified version of *cotH* was cloned into the vector pER19 [28], and the correct gene fusion verified by sequencing reactions. The resulting plasmid, pRG24, was introduced by single reciprocal (Campbell-like) recombination at the *cotH* locus of the *B. subtilis* chromosome. Several chloramphenicol-resistant clones were analyzed by PCR to select the clone containing the modified *cotH* promoter sequence upstream the entire *cotH* gene, yielding the strain RG24.

Double mutants lacking CotE or GerE and over-producing CotH (*pAH::cm*) were obtained by transforming competent cells of strains RH211 (*cotE*::spc) or KS450 (*gerE36*) (Suppl. Mat. Table S2) with chromosomal DNA extracted from strain RG24, yielding strains RG25 (*cotE::spc pAH::cm*) and RG26 (*gerE36 pAH::cm*), respectively.

Chromosomal DNA of strain DS127, carrying a functional *cotC::gfp* gene fusion [22], was used to transform competent cells of isogenic strains RH211 (*cotE::spc*) or AZ556 (*cotE::spc pAH::tet*) (Suppl. Mat. Table S2), yielding strains AZ569 and AZ571 respectively.

DNA fragments containing the 3′ terminal part of *cotA* (676 bp) or of *cotZ* (434 bp) were PCR amplified with oligonucleotides cotA-HindIII, cotA-PstI, cotZ-senso and cotZ-PstI (Suppl. Mat. Table S1). For *cotZ* fragment, an internal restriction site *Hind*III was used for cloning, yielding a fragment of 345 bp. Purified DNA fragments were then cloned in frame to the 5′ end of the *gfp* gene [22], yielding plasmids pTS33 and pTS32, respectively. Plasmids were used to transform competent cells of strain PY79 yielding strain AZ565 (*cotA::gfp*) and AZ573 (*cotZ::gfp*). Chromosomal DNA of the two strains was used to transform competent cells of isogenic strains RH211 (*cotE::spc*) or RG25 (*cotE::spc pAH::tet*) (Suppl. Mat. Table S2), yielding strains AZ570 (*cotA::gfp cotE::spc*), AZ574 (*cotZ::gfp cotE::spc*), AZ572 (*cotA::gfp cotE::spc pAH::tet*), AZ575 (*cotZ::gfp cotE::spc pAH::tet*).

### Transcriptional Analysis

Total RNA was extracted from sporulating cells 5, 7 and 9 hours after the onset of sporulation (T5, T7 and T9). For reverse transcription-PCR (RT-PCR) analysis a sample containing 2 µg of DNase-treated RNA was incubated with oligonucleotide H6 (Suppl. Mat. Table S1) at 65°C for 5 min and slowly cooled to room temperature to allow the primer annealing. RNAs were then retrotrascribed incubating the mixture at 50°C for 1 h in the presence of: 1 µl AffinityScript multi-temperature reverse transcriptase (Stratagene), 4 mM dNTPs, 1× reaction buffer (Stratagene), and 10 mM dithiothreitol (DTT). The enzyme was then inactivated at 70°C for 15 min. The obtained cDNA, was amplified by PCR, using primers CotApS and H (Suppl. Mat. Table S1) to analyze expression from *cotA* promoter, and with primers H12anti and H (Suppl. Mat. Table S1) to analyze the expression from *cotH* promoter. As a control, PCRs were carried out with RNA non-retrotranscribed to exclude the possibility that the amplification products could derive from contaminating genomic DNA.

### Sporulating Cells Lysates and Immunoblot Analysis

Sporulation of all *B. subtilis* strains was induced by the exhaustion method [29]. Sporulating cells were harvested at various times during sporulation and mother cells and forespore fractions were isolated as described before [21]. Whole-cell lysates of sporulating cells were prepared by sonication [21] followed by detergent treatment (62.5 mM Tris-HCl [pH 6.8], 4% SDS, 5% glycerol, 2% β-mercaptoethanol, 0.003% bromophenol blue) at 100°C for 7 min. 50 µg (mother cell extract or whole-cell lysates) or 20 µg (forespore extract) of total proteins was used for western blot analysis. Extraction of proteins from mature spores was performed with treatment at 65°C in SDS-DTT extraction buffer or at 4°C in 0.1 M NaOH [19]. Western blot analysis were performed by standard procedures. For electrotransfer was used nitrocellulose membrane and the proteins were then hybridated with either anti-CotH, anti-CotB, anti-CotG, anti-CotC or anti-CotA antibodies as described previously [21].

### Germination Efficiency and Lysozyme Resistance

Purified spores were heat activated as previously described [14] and diluted in 10 mM Tris-HCl (pH 8.0) buffer containing 1 mM glucose, 1 mM fructose, and 10 mM KCl. After 15 min at 37°C, germination was induced by adding 10 mML-alanine and the optical density at 580 nm was measured at 5-min intervals until a constant reading was reached.

Sensitivity to lysozyme was measured as described by Naclerio et al. [14]. Spores were prepared as previously described [29], omitting the lysozyme step and eliminating vegetative cells by heat treatment (10 min at 80°C). Purified spores were then suspended in 10 mM Tris-HCl (pH 7.0) buffer containing lysozyme (50 µg/ml), and the decrease in optical density was monitored at 595 nm at 1-min intervals for 10 min. Spore viability after lysozyme treatment was also measured following the CFUs on LB agar plates. Similar numbers of purified spores (between 3.0×10^4^ and 4.0×10^4^) of strains PY79, RH211 and RG25 were treated with lysozyme (50 µg/ml) for 3 minutes, diluted and plated for CFU determination.

### Microscopy

To prepare a culture for microscopy, 5 ml of Difco Sporulation (DS) medium was inoculated with a single colony of the strain, which was grown for 24 h at 37°C [22]. A 300 ml aliquot of cells were centrifuged for about 2 min in a microcentrifuge and resuspended in 10 µl of phosphate-buffered saline. 3 ml were placed on a microscope slide and covered with a coverslip previously treated for 30 s with poly-L-lysine (Sigma). Cells were observed with an Olympus BX41 fluorescence microscope. Typical acquisition times ranged from 400 to 1,000 ms for GFP and images were captured and cropped by using Analysis software.

### Surface-Enhanced Raman Scattering Spectroscopy

SERS spectroscopy analyses were performed by using a home-built system based on a Raman confocal microscope (Witec, alpha 300). The system is equipped with an excitation source at 532 nm, provided by a frequency doubled Nd:YAG. The silver colloids that formed the SERS-active substrate were prepared according to the standard procedure of Lee and Meisel [30], which is known to produce spherical Ag nanoparticles with an average diameter of 40 nm [31]. Briefly, AgNO_3_ (90 mg) was dissolved in 500 mL of H_2_O and brought to boil. A sodium citrate solution (10 mL, 1%) was added under vigorous stirring. The solution was kept boiling for 1 h. Before use, the silver solution was diluted appropriately with distilled water. To perform SERS measurements, an aliquot of the silver colloidal solution was added to an aqueous suspension of spores. Then the suspension was dipped on a glass microscope coverslip and left drying for 24 hours. SERS spectra were acquired with a Raman confocal microscope. For the measurements, a 60× objective was employed, providing an almost diffraction-limited spot on the sample. Raman spectra were acquired with a laser power of 52 µW and an integration time of 30 s.

## Supporting Information

Figure S1 **CotH is not part of the insoluble coat protein fraction.** Western blot of coat proteins extracted from mature spores of a wild type strain and isogenic strains lacking CotH (*cotH*) or CotE (*cotE*). Extraction was carried out either by standard SDS-DTT treatment [26] or by a decoating procedure developed to extract the insoluble coat protein fraction [15]. The amount of CotH extracted by the two methods is almost identical, suggesting that CotH is not part of the insoluble coat fraction. In strain *cotE*, CotH was not found confirming its dependence on CotE [13]. CotE was extracted in a slightly higher amount by the decoat method than by the standard SDS-DTT treatment, suggesting that it is partially insoluble.
(PPT)

Figure S2 **The CotE-dependent assembly of CotA is not affected by over-production of CotH.** A *cotA::gfp* fusion (AZ565) was introduced into isogenic strains lacking CotE (AZ570) or over-producing CotH in the absence of CotE (AZ572). A representative microscopy fields for each strain is shown by phase contrast and fluorescence (GFP) microscopy. Panels on the right are the merge of contrast phase and fluorescence images. Cultures were grown in DSM for 24 hours.
(PPT)

Table S1 **List of primers.**
(DOC)

Table S2 ***Bacillus subtilis*** **strains.**
(DOC)
