# Correction: Flexibility of the Programme of Spore Coat Formation in *Bacillus subtilis*: Bypass of CotE Requirement by Over-Production of CotH

**DOI:** 10.1371/annotation/cba64817-d823-47b0-b23c-3f07f51d6eec

**Published:** 2013-10-01

**Authors:** Rachele Isticato, Teja Sirec, Rosa Giglio, Loredana Baccigalupi, Giulia Rusciano, Giuseppe Pesce, Gianluigi Zito, Antonio Sasso, Maurilio De Felice, Ezio Ricca

There was an error in the title of the article.

The correct version of the title is: Flexibility of the Programme of Spore Coat Formation in *Bacillus subtilis*: Bypass of CotE Requirement by Over-Production of CotH

The correct citation is: Isticato R, Sirec T, Giglio R, Baccigalupi L, Rusciano G, et al. (2013) Flexibility of the Programme of Spore Coat Formation in *Bacillus subtilis*: Bypass of CotE Requirement by Over-Production of CotH. PLoS ONE 8(9): e74949. doi:10.1371/journal.pone.0074949 

