# Supplementary material for: Flexibility of the Prograamme of Spore Coat Formation in Bacillus subtilis: Bypass of CotE Requirement by Over-Production of CotH
Source: PLoS One. 2013 Sep 27;8(9):e74949. doi: 10.1371/journal.pone.0074949 (PMC3785510; doi:10.1371/journal.pone.0074949)
Supplement: Figure S1 — CotH is not part of the insoluble coat protein fraction. Western blot of coat proteins extracted from mature spores of a wild type strain and isogenic strains lacking CotH (cotH) or CotE (cotE). Extraction was carried out either by standard SDS-DTT treatment [26] or by a decoating procedure developed to extract the insoluble coat protein fraction [15]. The amount of CotH extracted by the two methods is almost identical, suggesting that CotH is not part of the insoluble coat fraction. In strain cotE, CotH was not found confirming its dependence on CotE [13]. CotE was extracted in a slightly higher amount by the decoat method than by the standard SDS-DTT treatment, suggesting that it is partially insoluble. (PPT) [file pone.0074949.s001.ppt]

## Slide 1
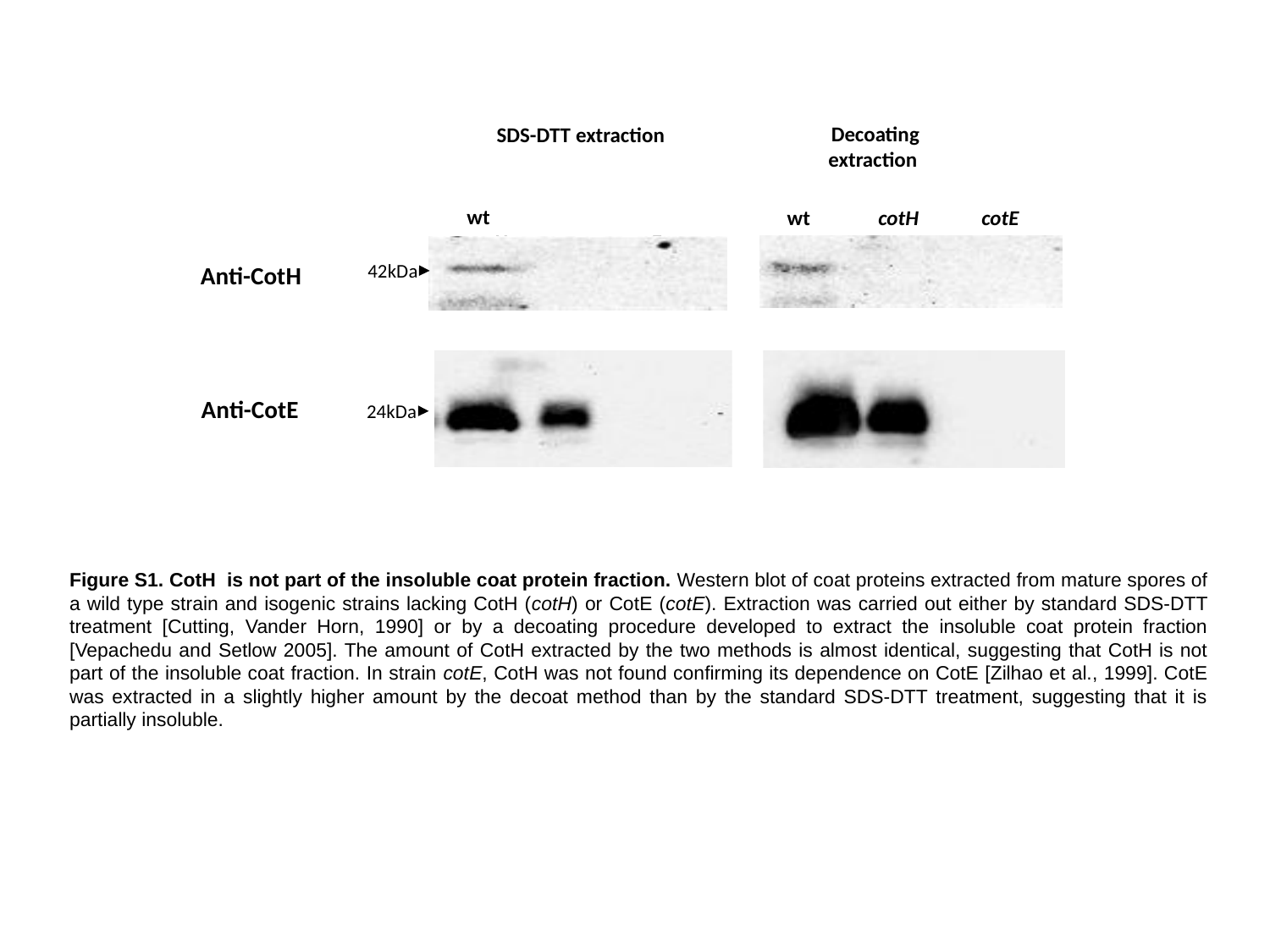

SDS-DTT extraction
Decoating
extraction
wt cotH cotE
wt cotH  cotE
42kDa
Anti-CotH
Anti-CotE
24kDa
Figure S1. CotH is not part of the insoluble coat protein fraction. Western blot of coat proteins extracted from mature spores of a wild type strain and isogenic strains lacking CotH (cotH) or CotE (cotE). Extraction was carried out either by standard SDS-DTT treatment [Cutting, Vander Horn, 1990] or by a decoating procedure developed to extract the insoluble coat protein fraction [Vepachedu and Setlow 2005]. The amount of CotH extracted by the two methods is almost identical, suggesting that CotH is not part of the insoluble coat fraction. In strain cotE, CotH was not found confirming its dependence on CotE [Zilhao et al., 1999]. CotE was extracted in a slightly higher amount by the decoat method than by the standard SDS-DTT treatment, suggesting that it is partially insoluble.
