# Supplementary material for: Flexibility of the Prograamme of Spore Coat Formation in Bacillus subtilis: Bypass of CotE Requirement by Over-Production of CotH
Source: PLoS One. 2013 Sep 27;8(9):e74949. doi: 10.1371/journal.pone.0074949 (PMC3785510; doi:10.1371/journal.pone.0074949)
Supplement: Figure S2 — The CotE-dependent assembly of CotA is not affected by over-production of CotH. A cotA::gfp fusion (AZ565) was introduced into isogenic strains lacking CotE (AZ570) or over-producing CotH in the absence of CotE (AZ572). A representative microscopy fields for each strain is shown by phase contrast and fluorescence (GFP) microscopy. Panels on the right are the merge of contrast phase and fluorescence images. Cultures were grown in DSM for 24 hours. (PPT) [file pone.0074949.s002.ppt]

## Slide 1
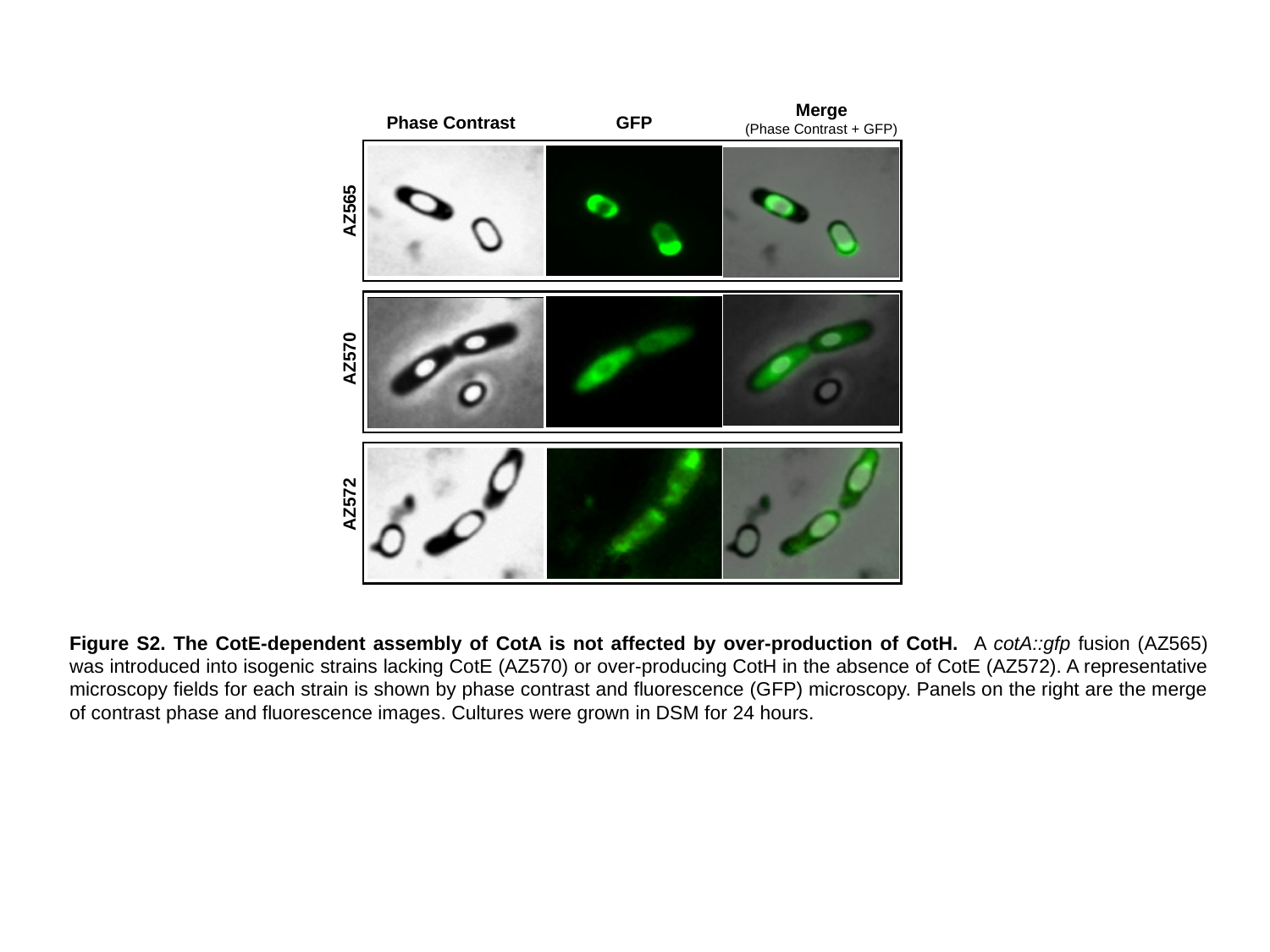

Merge
(Phase Contrast + GFP)
Phase Contrast
GFP
AZ565
AZ570
AZ572
Figure S2. The CotE-dependent assembly of CotA is not affected by over-production of CotH. A cotA::gfp fusion (AZ565) was introduced into isogenic strains lacking CotE (AZ570) or over-producing CotH in the absence of CotE (AZ572). A representative microscopy fields for each strain is shown by phase contrast and fluorescence (GFP) microscopy. Panels on the right are the merge of contrast phase and fluorescence images. Cultures were grown in DSM for 24 hours.
