# Supplementary material for: Flexibility of the Prograamme of Spore Coat Formation in Bacillus subtilis: Bypass of CotE Requirement by Over-Production of CotH
Source: PLoS One. 2013 Sep 27;8(9):e74949. doi: 10.1371/journal.pone.0074949 (PMC3785510; doi:10.1371/journal.pone.0074949)
Supplement: Table S1 — List of primers. (DOC) [file pone.0074949.s003.doc]

**Table S1.** List of primers.

| *Primer* | *Sequence^a^* |
| --- | --- |
| H32s | AATAAAAGCCTAAAATTTGTAAGGAGGATTATGG |
| H | CGCggatccGCGCCGgaattcAGCGATATCAATATCCAG |
| H13 | TCCTTGAATTCTTACATAATCG |
| H12anti | GTATAAGTGTTCCGCTGAGATCTT |
| H6 | CTCTGATATGTGATCCCC |
| CotApS | CGTCTATCTTGTCATCGCC |
| CotAp3a | CAAATTTTAGGCTTTTATTTACTATAGTTAATGACAATAAGG |
| cotZ-senso | gaattcAAACATCAAGCTGCGTGCGT |
| cotZ-PstI | ctgcagATGATGATGTGTACGATTGA |
| cotA-HindIII | aagcttACTGAATTCTTTCAGCC |
| cotA-PstI | ctgcagTTTATGGGGATCAGTTA |

^a^ Underlined letters in primer H32s indicate nucleotides complementary to the underlined sequence of primer CotAp3a. Not capital letters indicate restriction recognition sites.
