# Supplementary material for: Flexibility of the Prograamme of Spore Coat Formation in Bacillus subtilis: Bypass of CotE Requirement by Over-Production of CotH
Source: PLoS One. 2013 Sep 27;8(9):e74949. doi: 10.1371/journal.pone.0074949 (PMC3785510; doi:10.1371/journal.pone.0074949)
Supplement: Table S2 — Bacillus subtilis strains. (DOC) [file pone.0074949.s004.doc]

**Table S2**. *Bacillus subtilis* strains.

| **Strain** | **Genotype** | **Source** |
| --- | --- | --- |
| PY79 | wild type | [25] |
| RH211 | *cotE::spc* | [21] |
| BZ213 | *cotE::cm* | [7] |
| ER220 | *cotH::spc* | [14] |
| RG24 | *pAH::cm* | This study |
| RG25 | *cotE::spc pAH::cm* | This study |
| RG26 | *gerE36 pAH::cm* | This study |
| AZ569 | *cotE::spc cotC::gfp* | This study |
| AZ571 | *cotE::spc pAH::cm cotC::gfp* | This study |
| AZ565 | *cotA::gfp* | This study |
| AZ570 | *cotE::spc cotA::gfp* | This study |
| AZ572 | *cotE::spc pAH::cm cotA::gfp* | This study |
| AZ573 | *cotZ::gfp* | This study |
| AZ574 | *cotE::spc cotZ::gfp* | This study |
| AZ575 | *cotE::spc pAH::cm cotZ::gfp* | This study |
| KS450 | *gerE36* | [7] |
| DS127 | *cotC::gfp* | [22] |
